# Dynamical Systems on Generalised Klein Bottles

**DOI:** 10.3390/e27020119

**Published:** 2025-01-24

**Authors:** Peter Grindrod, Ka Man Yim

**Affiliations:** 1 Mathematical Institute, University of Oxford, Oxford OX1 2JD, UK; 2 School of Mathematics, Cardiff University, Cardiff CF24 4AG, UK; yimkm@cardiff.ac.uk

**Keywords:** generalised Klein bottles, distributions and flows with Klein bottle symmetries, information processing within mammalian brains, topological data analysis

## Abstract

We propose a high-dimensional generalisation of the standard Klein bottle extending beyond those considered previously. We address the problem of generating continuous scalar fields (distributions) and dynamical systems (flows) on such state spaces, which can provide a rich source of examples for future investigations. We consider a class of high-dimensional dynamical systems that model distributed information processing within the human cortex, which may be capable of exhibiting some Klein bottle symmetries. We deploy topological data analytic methods in order to analyse their resulting dynamical behaviour and suggesting future challenges.

## 1. Introduction

Data science typically presents observations (in the form of data) from some implied dynamical system of interest and demands that we character the underlying relevant processes and system behaviour. This includes differentiating one source from another, and other related questions regarding their observed state variables’ mutual dependencies and mutual information.

Here, we consider the problem of characterising finite dimensional manifolds that contain such attractors. The most basic problem is to consider a wide range of the possible finite dimensional manifolds which may be present. This leads us naturally to ask what kind of compact closed manifolds exist and whether we may recognise scalar fields (smooth distributions and potentials) or vector fields (flows) flows defined over them, respecting their inherent topologies.

For low-dimensional state spaces, there is a limited set of possible compact closed manifolds. In two dimensions, for example, we have the two-sphere, the two-torus, the Klein bottle and the real projective plane. In higher dimensions, these manifolds may generalise to other objects. Generalising spheres and tori is straightforward.

In this paper, we introduce a generalisation of the Klein bottle, extending beyond those given previously [1]. In *k* dimensions, this relies on a partition of independent coordinates into $k_{1}$ periodic coordinates that may be flipped or swapped (as if from moving clockwise to moving anticlockwise, for example) and $k_{2}$ active periodic components which cause the various flips (such that $k=k_{1}+k_{2}$).

By doing so, we access a wide range of compact closed manifolds over which we may define, or there may be discovered, dynamical systems.

We introduce an inverse problem in the form of the observation of a large-scale spiking system, including those carrying out information processing within the cortex of the human brain [2], where the dimension of the attractor may be estimated yet the topological nature of the attractor is elusive. Such problems may become common as high-throughput science enables the complete observation of such large-scale dynamical systems which appear to approach unknown attractors of relatively high dimension that require characterisation. Working from observed time series, such as the spike trains as here, requires an analytical pipeline. The very high state space dimension of such a complex system means that the dimension and topology of the manifold holding the attractor is essential in gaining an understanding of *what is occurring* and *how the system responds* to incoming stimuli.

The detailed and rigorous mathematical considerations are deferred to Appendices.

## 2. Generalised Klein Bottles

For $k_{1}\geq 1$ and $k_{2}\geq 0$, we let $B=(B_{i,j})$ denote an irreducible binary $k_{1}\times k_{2}$ matrix.

We consider $R^{k_{1}+k_{2}}$ with independent coordinates, $x={\left(x_{1},x_{2}\ldots ,x_{k_{1}}\right)}^{T}$ and $y={\left(y_{1},y_{2}\ldots ,y_{k_{2}}\right)}^{T}$, subject to the following set of symmetries:

(1) $$\left(x+a,y+b\right)∼\left(\varphi \left(b\right)x,y\right)),\;\;\left(a,b\right)\in Z^{k_{1}}\times Z^{k_{2}},$$

where $\varphi \left(b\right):Z^{k_{2}}\to \mathrm{Aut}\left(Z^{k_{1}}\right)$ is a *b*-dependent automorphism defined by the Hadamard (elementwise) product

$$\varphi \left(b\right)x={\left(-1\right)}^{B.b}⊙x=\left({\left(-1\right)}^{{\left(B.b\right)}_{1}}x_{1},\ldots ,{\left(-1\right)}^{{\left(B.b\right)}_{k_{1}}}x_{k_{1}}\right)$$

and extended to $R^{k_{1}}$. So, depending on the parity of the *i*th term of the vector ${\left(-1\right)}^{B.b}$, the sign of the *i*th coordinate, $x_{i}$, is either flipped (reflected) or else left unchanged.

The symmetries in (1) imply the quotient space is the cube, ${\left[0,1\right]}^{k_{1}+k_{2}}$, with no boundaries and endowed locally with the Euclidean metric. We refer to the $x_{i}$ coordinates as *toroidal coordinates*, since they are 1-periodic. We refer to the $y_{j}$ coordinates as *Klein coordinates* since they are one-periodic and when incremented by one they also flip, or reflect, some of the $x_{i}$ toroidal coordinates (those for which $B_{i,j}=1$). The result is a **generalised Klein bottle**, which we denote by $K(k_{1},k_{2},B)$.

**Figure 1:**
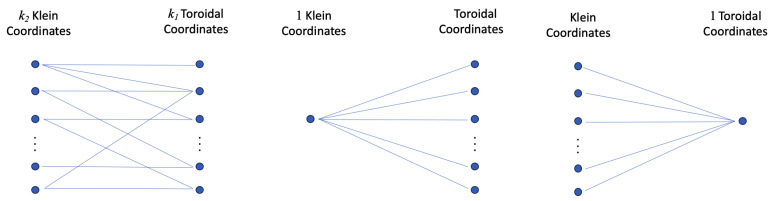
Left: Space $K(k_{1},k_{2},B)$ is specified by a bipartite graph indicating which of the $k_{1}$ toroidal coordinates is flipped by which of the $k_{2}$ Klein coordinates. It must be strongly connected (irreducible). Centre: $K(k_{1},1,B)$ as defined in [1] (with only one possibility for *B*). Right: $K(1,k_{2},B)$ (again with only one possibility for *B*).

*B* specifies a bipartite graph indicating which of the toroidal coordinates are flipped by which of the Klein coordinates; see Figure 1. The adjacency matrix, *A*, for the associated bipartite graph is given in block matrix form by

$$A=\left(\begin{array}{cc} 0 & B\\ B^{T} & 0 \end{array}\right).$$

Since we have assumed *B* is irreducible, the corresponding bipartite graph cannot be decomposed into disjoint connected graphs. Necessarily, every Klein coordinate flips at least one toroidal coordinate, and every toroidal coordinate is flipped by at least one Klein coordinate (for if this were not the case, any marooned coordinates could be discarded).

If $k_{1}=k_{2}=1$, then we have the standard Klein bottle (B=(1) in that case), a closed non-orientable two-dimensional surface. If $k_{2}=0$, then $K\left(k_{1},0\right)=T^{k_{1}}$ the $k_{1}$-dimensional torus. For spaces $K(1,k_{2},B)$, for $k_{2}\geq 2$, there is but one valid choice of bipartite graph, *B*. Those spaces are distinct from the Klein bottle generalisations previously considered in [1] (and its descendants), which are $K(k_{1},1,B)$, for some $k_{1}\geq 2$, where again there is but one valid choice for *B*; see Figure 1.

When two Klein variables only affect the same toroidal variable, there is a *hidden* torus. We let $e_{i}$ denote the unit vectors in the direction of the *i*th Klein coordinate in $R^{k_{2}}$. We consider, for example, K(1,n,B) for n≥1. For any distinct pair of the *n* Klein coordinate symmetries, we say those in $y_{i}$ and $y_{j}$ (where without loss of generality i<j), we deduce

$$\begin{array}{c} \left(x,\ldots y_{i}+1,\ldots ,y_{j}+1,\ldots \right)∼\left(x,\ldots y_{i},\ldots ,y_{j},\ldots \right),\\ \left(x,\ldots y_{i}-1,\ldots ,y_{j}+1,\ldots \right)∼\left(x,\ldots y_{i},\ldots ,y_{j},\ldots \right). \end{array}$$

Hence, there is a two-dimensional torus, $T^{2},$ with coordinate directions $\left(\pm e_{i}+e_{j}\right)/\sqrt{2}$, and periodicities $\sqrt{2}$, within the $\left(y_{i},y_{j}\right)$-subspace of K(1,n,B).

An inference of the this type is true more generally within $K(k_{1},k_{2},B)$, provided that the subsets of the toroidal coordinates that are flipped by each of a pair $y_{i}$ and $y_{j}$ (and hence the *i* and *j*th columns of *B*) are identical. Such a situation might occur in scenarios where there is some symmetry within the application, meaning that some of that state variables which are represented by Klein coordinates are permutable (exchangeable) and thus may cause similar effects (transformations of finite order) on a common subset of the toroidal coordinates. More generally, this type of hidden symmetry is a consequence of the dimension of the range of *B*, spanned by its columns, and is strictly less than $k_{2}$.

## 3. More Symmetries

For the general case of $K(k_{1},k_{2},B)$, we rewrite this to consider $B\in Z^{k_{1}\times k_{2}}$ (since only parity of the elements of *B* matter), and also set $\varphi :Z^{k_{2}}\to \mathrm{Aut}\left(Z^{k_{1}}\right)$, extended to be defined on $R^{k_{1}}$, via matrix multiplication

(2) $$\varphi \left(b\right)x=H\left(b\right).x\;\;\mathrm{for}\;b\in Z^{k_{2}}\;\;\mathrm{and}\;\mathrm{all}\;x\in R^{k_{1}},$$

where we introduce the diagonal matrix (containing diagonal elements in {−1,1}) given by

(3) $$H\left(b\right)\equiv \mathrm{Diag}\{{\left(-1\right)}^{B.b}\}$$

Then, $H\left(b\right).x\equiv {\left(-1\right)}^{B.b}⊙x$ (as before).

So far φ is subordinate to the choice of *B*. Yet it might be generalised further.

We consider a group automorphism $\varphi :Z^{k_{2}}\to \mathrm{Aut}\left(Z^{k_{1}}\right)$ whose image subgroup in $\mathrm{Aut}(Z^{k_{1}})$ is of finite order. The automorphism group $\mathrm{Aut}(Z^{k_{1}})$ is the general linear group $\mathrm{GL}(k_{1};Z)$; that is, the group of $k_{1}\times k_{1}$ matrices with integer entries and determinant ±1. The image of φ is an abelian subgroup of $\mathrm{GL}(k_{1};Z)$, and the homomorphism can be represented by picking $k_{2}$ matrices $M_{1},\ldots ,M_{k_{2}}\in \mathrm{GL}\left(k_{1},Z\right)$ that commute with each other:

(4) $$\varphi \left(b\right)=H\left(b\right)\;\;\mathrm{where}\;H\left(b\right)=M_{1}^{b_{1}}.\cdots M_{k_{2}}^{b_{k_{2}}}.$$

In the diagonal special case for (4), given by (2) and (3) and leading to the space $K(k_{1},k_{2},B)$, we have

(5) $$H\left(b\right)=D_{1}^{b_{1}}.\cdots .D_{k_{2}}^{b_{k_{2}}},$$

where each $D_{i}$ is a diagonal matrix with a sequence of ±1s down the diagonal. Equation (5) represents the natural factorisation of H(b) as given in (3).

We let $K(k_{1},k_{2};\varphi )$ denote the space defined by $R^{k_{1}}\times R^{k_{2}}$ modulo the following equivalence relation: for any pair of integers $\left(a,b\right)\in Z^{k_{1}}\times Z^{k_{2}}$,

(6) (x+a,y+b)∼(φ(b)x+a,y+b).

This equivalence relation is in fact the quotient of $R^{k_{1}}\times R^{k_{2}}$ by action of the semi-direct product $G=Z^{k_{1}}{⋊}_{\varphi }Z^{k_{2}}$, on which multiplication is given by

$$\left(a^{\prime },b^{\prime }\right)⋄\left(a,b\right)∼\left(\varphi \left(b^{\prime }\right)a+a^{\prime },b+b^{\prime }\right).$$

The group $Z^{k_{1}}{⋊}_{\varphi }Z^{k_{2}}$ acts on $R^{k_{1}}\times R^{k_{2}}$ in the following manner:

(7) (a,b)·(x,y)=(φ(b)x+a,y+b).

One can easily verify this action is compatible with the group multiplication ⋄.

We make some observations about the group action. The action is free: (a,b)·(x,y)=(x,y) if and only if (a,b)=(0,0), the identity element in *G*. Furthermore, because any non-identity element of *G* changes in the coordinates of (x,y) by a non-zero, integer amount, the action is a covering space action on $R^{k_{1}}\times R^{k_{2}}$, and the covering of $R^{k_{1}}\times R^{k_{2}}$ of K is a regular covering.

**Example** **1.** *We give an example where the image of φ in (4) is not the subgroup of diagonal matrices. For $k_{1}=2$ and $k_{2}=1$, we can have the following homomorphism: φ:Z→GL(2,Z), where*

(8) $$\varphi \left(b\right)x\equiv H\left(b\right).x\;\;\mathrm{where}\;H\left(b\right)={\left(\begin{array}{cc} 0 & 1\\ 1 & 0 \end{array}\right)}^{b}.$$

*The action of $\left(a_{1},a_{2},b\right)$ on $\left(x_{1},x_{2},y\right)$ is then given by*

(9) $$\left(\begin{array}{c} x_{1}\\ x_{2}\\ y \end{array}\right)\mapsto \left(\begin{array}{c} {\left(\begin{array}{cc} 0 & 1\\ 1 & 0 \end{array}\right)}^{b}\left(\begin{array}{c} x_{1}\\ x_{2} \end{array}\right)\\ y \end{array}\right)+\left(\begin{array}{c} a_{1}\\ a_{2}\\ b \end{array}\right)$$

*We note that in this case the parity of b controls whether the $x_{1}$ and $x_{2}$ coordinates are swapped (i.e., a reflection across the diagonal of the first two components), whereas previously it controlled the reflection of one or more component coordinates.*

### 3.1. Reduction of Symmetries

Since the equivalence relations on $R^{k_{1}}\times R^{k_{2}}$ are due to a quotient by a group action, it suffices to write down the equivalence relations generated by the generators of the group, as they imply all of the other equivalence relations. Since the group $Z^{k_{1}}{⋊}_{\varphi }Z^{k_{2}}$ is generated by the set $\left\{\left(e_{i},0\right),\left(0,e_{j}\right)\right\}$ where $e_{i}$s and $e_{j}$s are unit coordinate vectors in $Z^{k_{1}}$ and $Z^{k_{2}}$, respectively, we can express the equivalence relations in Equation (6) as follows: for all $\left(x,y\right)\in R^{k_{1}}\times R^{k_{2}}$,

(10) $$\left(x,y\right)∼\left(x+e_{i},y\right)\;\mathrm{and}\;\left(x,y\right)∼\left(\varphi \left(e_{j}\right)x,y+e_{j}\right).$$

We can also choose the set of generators to be those that generate the kernel and co-image of φ. We consider in particular the diagonal case Equation (3), where

$$\begin{array}{c} \varphi \left(b\right)=\mathrm{Diag}\{{\left(-1\right)}^{B.b}\}. \end{array}$$

Regardless of the choice of *B*, if $b\in {\left(2Z\right)}^{k_{2}}$, then φ(b)=1. We now analyse how the choice of *B* affects the action. Since ${\left(-1\right)}^{2(B.b)}=1$ as well, we can trivially write

$$\begin{array}{c} \varphi \left(b\right)=\mathrm{Diag}\{{\left(-1\right)}^{(B.(b\;\mathrm{mod}\;2))\;\mathrm{mod}\;2}\}. \end{array}$$

In other words, we regard the binary matrix *B* as a linear transformation of $Z_{2}$-vector spaces. The image of φ are $k_{1}$-dimensional diagonal matrices with ±1 entries. The set of such matrices forms a subgroup of $\mathrm{GL}(k_{1},Z)$ isomorphic to $Z_{2}^{k_{1}}$. The isomorphism is given by

(11) $$\begin{array}{c} \exp:c\in Z_{2}^{k_{1}}\mapsto \mathrm{Diag}\left\{{\left(-1\right)}^{c}\right\}. \end{array}$$

Thus, we can express φ as the composition that factors through

(12) $$\varphi :Z^{k_{2}}\overset{\mathrm{mod}\;2}{\to }Z_{2}^{k_{2}}\overset{B}{\to }Z_{2}^{k_{1}}\overset{\exp}{\underset{≅}{\to }}Z_{2}^{k_{1}}.$$

The kernel kerφ consists of even integers and integers (mod 2) in the kernel of *B*; the image of φ is isomorphic to the image of *B* in $Z_{2}^{k_{1}}$. Computationally, the basis vectors of the kernel and image of *B* can be easily inferred by performing Gaussian elimination on matrices derived from *B* over the field $Z_{2}$. We let $r\leq k_{2}$ be the rank of *B*, and suppose columns $j_{1},\ldots ,j_{r}$ of *B* form a basis for the image of *B*. We can express the equivalence relations as

(13) $$\left(x,y)∼(x+e_{i},y\right)$$

(14) $$\left(x,y)∼(x,y+2e_{j}\right)$$

(15) (x,y)∼(x,y+c)   c∈kerB

(16) $$(x,y)∼({\left(-1\right)}^{B_{j_{k}}}⊙x,y+e_{j_{k}})\;\;\;k=1,\ldots ,r.$$

We note that *c* is a vector in $Z_{2}^{k_{2}}$, realised as a real vector with {0,1} entries in $R^{k_{2}}$. The first two symmetry conditions is a *toroidal* symmetry on the whole space. The latter two equivalence relations can be expressed as a $Z_{2}^{k_{2}}$ action on the torus obtained by imposing the first two equivalence relations on $R^{k_{1}}\times R^{k_{2}}$. We expound on this point of view in Appendix A.

**Example** **2.** *We consider the full coupling case, where B is the $k_{1}\times k_{2}$ binary matrix with entries all equal to one. The kernel of B is spanned by $\left(k_{2}-1\right)$$Z_{2}$-vectors*

(1,1,0,…,0),(0,1,1,0,…,0),…,(0,…,1,1),

*and the image of B is simply vector (1,…,1). Thus, the equivalence relations are generated by*

$$\begin{array}{cc} \left(x,y\right) & ∼(x+e_{i},y)\\ \left(x,y\right) & ∼(x,y+2e_{j})\\ \left(x,y\right) & ∼\left(x,y+e_{i}+e_{i+1}\right)\;\;\;i=1,\ldots ,k_{2}-1\\ \left(x,y\right) & ∼(-x,y+e_{1}). \end{array}$$

### 3.2. Functions on Klein Bottles

We can specify scalar functions on $K(k_{1},k_{2},B)$ uniquely with functions $F:R^{k_{1}+k_{2}}\to R$ that satisfy condition

(17) F(x,y)=F((a,b)·(x,y))=F(φ(b)x+a,y+b),

for all $\left(a,b\right)\in G=Z^{k_{1}}{⋊}_{\varphi }Z_{k_{2}}$. Because $K(k_{1},k_{2},B)$ is a quotient of $q:R^{k_{1}+k_{2}}↠K\left(k_{1},k_{2}\right)$, any function f:K→R admits a lift $F=f\circ q:R^{k_{1}+k_{2}}\to R$ to a function on the covering space. Alternatively, since $K(k_{1},k_{2})$ is the orbit space of the group action of $Z^{k_{1}}⋊Z^{k_{2}}$ on $R^{k_{1}+k_{2}}$, any function *F* that is constant on the orbits descends to function *f* on $K(k_{1},k_{2})$, such that F=f∘q.

We begin by demonstrating a simple, practical method of construction for a wide range of functions, *F*, with the desired properties.

We consider $K(k_{1},k_{2},B)$, as before. Suppose that we for each $i=1,\ldots ,k_{1}$ have two non-negative smooth functions, $T_{i,1}\left(y\right)$ and $T_{i,2}\left(y\right)$, defined for $y\in R^{k_{2}}$, such that

$$T_{i,2}\left(0\right)=1\;\;\mathrm{and}\;\;T_{i,2}\left(0\right)=0,$$

and if $B_{i,k}=1$, then $T_{i,1}\left(y\right)$ and $T_{i,2}\left(y\right)$ exchange their values each time $y_{k}$ is incremented by one. Clearly, $T_{i,1}\left(y\right)$ and $T_{i,2}\left(y\right)$ must be two-periodic in all arguments.

Then, for $b\in Z^{k_{2}}$, we have

$$\begin{array}{c} \left(T_{i,1}\left(y+b\right),T_{i,2}\left(y+b\right)\right)=\left\{\begin{array}{c} \left(T_{i,1}\left(y\right),T_{i,2}\left(y\right)\right)\;\;\mathrm{if}\;{\left(B.b\right)}_{i}\;\mathrm{is}\;\mathrm{even},\\ \left(T_{i,2}\left(y\right),T_{i,1}\left(y\right)\right)\;\;\mathrm{if}\;{\left(B.b\right)}_{i}\;\mathrm{is}\;\mathrm{odd}. \end{array}\right. \end{array}$$

We consider the following ansatz:

(18) $$F\left(x,y\right)=\prod_{i=1}^{k_{1}}\left(T_{i,1}\left(y\right)f_{i}\left(x_{i}\right)+T_{i,2}\left(y\right)f_{i}\left(1-x_{i}\right)\right)\;$$

Here, the $f_{i}\left(x_{i}\right)$, for $i=1,\ldots ,k_{1}$, are arbitrary smooth one-periodic functions, and the pair $T_{i,1}\left(y\right)$ and $T_{i,2}\left(y\right)$ switch, or interpolate, the corresponding term in the product between $f_{i}\left(x_{i}\right)$ and its flipped form $f_{i}\left(1-x_{i}\right)$ as the $y_{k}$s are incremented. Hence, being a constant along orbit, F(x,y) descends to a function defined on $K(k_{1},k_{2},B)$. It is separable, and such that, at y=0, we have $F\left(x,0\right)=\prod_{j=1}^{k_{1}}f_{j}\left(x_{j}\right).$

The switching functions, $T_{i,1}\left(y\right)$, and $T_{i,2}\left(y\right)$, depend on *B* and may be defined as follows. First, we consider $K(k_{1},k_{2},B)$ and define function $S_{i}\left(y\right)$ depending on the *i*th row of *B*:

$$S_{i}\left(y\right)\equiv \prod_{k=1}^{k_{2}}{\left(\frac{-(1-\cos\pi y_{k})}{2}+\frac{\left(1+\cos\pi y_{k}\right)}{2}\right)}^{B_{i,k}},\;\;i=1,\ldots ,k_{1}$$

This product of the sum expression for $S_{i}$ may be expanded out as a sum of product terms, with each term containing a product of exactly $k_{2}$ factors. Then, we define $T_{i,1}\left(y\right)$ to be the sum of all the positive terms within that sum (those terms having an even number of negative factors each of the form $\left\{-\left(1-\cos\pi y_{k}\right)\right\}$ for which $B_{i,k}=1$), and $-T_{i,2}\left(y\right)$ to be the sum of all the negative terms within that sum (those terms having an odd number of negative factors each of the form $\left\{-\left(1-\cos\pi y_{k}\right)\right\}$ for which $B_{i,k}=1$). Then, by definition, $T_{i,1}$, and $T_{i,2}$ are two-periodic in all of their arguments, and we have

$$S_{i}\left(y\right)=T_{i,1}\left(y\right)-T_{i,2}\left(y\right),$$

expressing $S_{i}$ as the difference between two non-negative continuous functions, where $S_{I}\left(0\right)=T_{i,1}\left(0\right)=1$ and $T_{i,2}\left(0\right)=0$.

Note that we may also write

$$S_{i}\left(y\right)=\prod_{k=1}^{k_{2}}{\left(\cos\pi y_{k}\right)}^{B_{i,k}}.$$

Hence, when each of the $y_{k}$s for which $B_{i,k}=1$ are independently incremented by one, $S_{i}$ must change sign, and consequently $T_{i,1}$ and $T_{i,2}$ must exchange their values. The pair thus subsumes the Klein symmetries of $K(k_{1},k_{2},B)$.

Defined in this way or otherwise, the pair $T_{i,1}$ and $T_{i,2}$ implies that function *F*, given by the ansatz in (18), descends to a scalar field on $K(k_{1},k_{2},B)$, as required.

In Appendix A, we consider the necessary and sufficient conditions for scalar fields satisfying the symmetry condition F(z)=F(g·z) and derive a Fourier basis for such scalar fields. The condition is expressed as *F* being in the kernel of a linear operator L on scalar fields, which we describe in Equation (A6). This operator can be interpreted as a graph Laplacian; the technical details are briefly summarised in Remark A3.

Using the Fourier transform (which is natural given the periodicities), the symmetry requirements imposed on *F* imply constraints on the Fourier coefficients of *F*. These constraints are expressed, once again, linearly: the coefficients are in the kernel of operator $L^{★}$ on the vector space of Fourier coefficients, which is dual to L (Equation (A7)). By taking the inverse Fourier transform on coefficients in the kernel of $L^{★}$, we can find a Fourier basis for scalar functions that satisfy the symmetry constraints.

**Figure 2:**
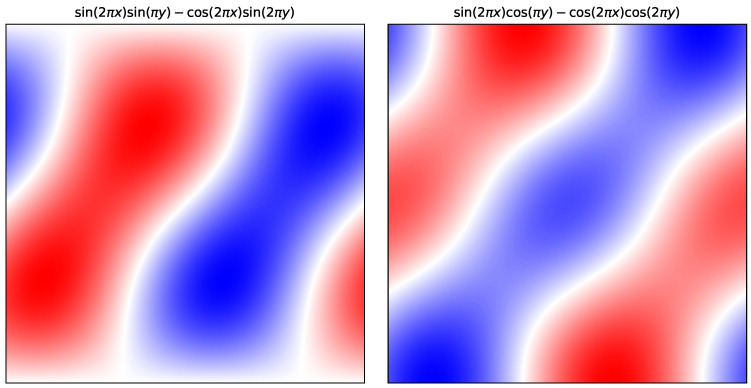
Examples of two scalar fields on the standard Klein bottle K(1,1). The Klein bottle is illustrated here as a unit square domain where the top and bottom sides are identified with opposite orientation, and the left and right hand sides are identified with the same orientation. The scalar fields are written as linear combinations of Fourier bases functions compatible with the group action on $R^{2}$ that has the Klein bottle as the quotient. The heat maps of these functions are coloured such that white indicates where the function is zero; the colour scale from red to white then blue moves from positive to negative.

**Example** **3.** *We plot some Fourier modes of scalar fields on the standard Klein bottle in Figure 2 as an illustration. These are formed by linear combinations of Fourier basis functions of the form*

$$\begin{array}{cc} \cos(2\pi \lambda x)\cos(\pi \zeta y+\phi )\; & \;\lambda \in Z_{\geq 0},\zeta \;even\\ \sin(2\pi \lambda x)\cos(\pi \zeta y+\phi )\; & \;\lambda \in Z_{\geq 0},\zeta \;odd. \end{array}$$

*These are derived in Example A4 in Appendix A. We can verify that these functions satisfy the symmetry conditions: for integers a,b, if ζ is even,*

$$\begin{array}{cc} \cos\left(2\pi \lambda \left({\left(-1\right)}^{b}x+a\right)\right)\cos\left(\pi \zeta \left(y+b\right)+\phi \right) & =\cos(2\pi \lambda x)\cos(\pi \zeta y+\phi ). \end{array}$$

*On the other hand, if ζ is odd,*

$$\begin{array}{cc} \sin\left(2\pi \lambda \left({\left(-1\right)}^{b}x+a\right)\right)\cos\left(\pi \zeta \left(y+b\right)+\phi \right) & ={\left(-1\right)}^{b(\zeta +1)}\sin\left(2\pi \lambda x\right)\cos\left(\pi \zeta y+\phi \right)\\ & =\sin(2\pi \lambda x)\cos(\pi \zeta y+\phi ). \end{array}$$

*The calculations in Example A4 show that these functions form a basis for any function admitting a Fourier transform while satisfying the symmetry conditions for the Klein bottle.*

### 3.3. Vector Fields on Klein Bottles

In this section, we offer a parameterisation for continuous vector fields on the Klein bottle, $K(k_{1},k_{2},B)$. Unlike Euclidean space, where vector fields can be expressed by any smooth maps $R^{d}$ to $R^{d}$, on a Klein bottle we can transport a local coordinate frame around one of the non-contractible loops of $K(k_{1},k_{2},B)$ that flips it. In concrete terms, the non-orientability prevents us from expressing vectors at different points on K with a consistently defined set of global coordinates: we require a flip. In contrast, on the flat torus we can indeed parallel transport a local coordinate frame from one point to any other point and express vectors at different locations with the a coordinate system that is periodic in each toroidal coordinate.

However, we can lift (pull back) vectors on the Klein bottle to its universal covering space $R^{k_{1}+k_{2}}$ where we have a global (x,y) coordinate frame. By matching the origin of $R^{k_{1}+k_{2}}$ and the coordinate system to the local coordinate system of a point in $K(k_{1},k_{2},B)$, the explicit coordinate expression of the vector field lifted in $R^{k_{1}+k_{2}}$ should reflect the change in the orientation of the coordinate system in K. We derive the following symmetry condition on lifted vector fields in Appendix B. Writing the lifted vector field *V* in components V=(X,Y) where $X\in R^{k_{1}}$ and $Y\in R^{k_{2}}$, we have, for $\left(a,b\right)\in Z^{k_{1}}⋊Z^{k_{2}}$,

(19) $${\left(-1\right)}^{B.b}⊙X\left(x,y\right)=X({\left(-1\right)}^{B.b}⊙x+a,y+b)$$

(20) $$Y(x,y)=Y({\left(-1\right)}^{B.b}⊙x+a,y+b).$$

We begin by demonstrating this possibility via a simplifying ansatz (that subsumes the required Klein symmetries above). We provide a more exhaustive and abstract approach in Appendix B.

First, we consider $K(k_{1},k_{2},B)$ and let $T_{i,1}\left(y\right)$ and $T_{i,1}\left(y\right)$ be defined as before, in Section 3.2.

Then, we consider the following ansatz:

(21) $$X_{i}\left(x,y\right)=\left(T_{i,1}\left(y\right)f_{i,i}\left(x_{i}\right)-T_{i,2}\left(y\right)f_{i,i}\left(1-x_{i}\right)\right)\prod_{j=1\;j\neq i}^{k_{1}}\left(T_{j,1}\left(y\right)f_{i,j}\left(x_{j}\right)+T_{j,2}\left(y\right)f_{i,j}\left(1-x_{j}\right)\right)$$

for $i=1,\ldots ,k_{1};$

(22) $$Y_{i}\left(x,y\right)=\prod_{j=1}^{k_{1}}\left(T_{j,1}\left(y\right)g_{i,j}\left(x_{j}\right)+T_{j,2}\left(y\right)g_{i,j}\left(1-x_{j}\right)\right)\;$$

for $i=1,\ldots ,k_{2}.$

The form in (21) allows for any of the $x_{j}$-coordinate dependencies of $X_{i}$ to be reversed by suitably incrementing the appropriate *y*-coordinates, and it also induces a change in sign in $X_{i}$ whenever $x_{i}$ is reversed. The form in (22) allows for the $x_{j}$-coordinate dependencies of $Y_{i}$ to be reversed by suitably incrementing the appropriate *y*-coordinates.

The vector field given in (21) and (22) respects the appropriate symmetries for $K(k_{1},k_{2},B)$, which are inherited from the fact of the swapping of $T_{i,1}$ and $T_{i,2}$ values as appropriate coordinates $y_{k}$ are incremented by one. Hence, it defines a separable vector field over $K(k_{1},k_{2},B)$.

We can, of course, linearly combine similar separable fields to obtain more general flows on $K(k_{1},k_{2},B)$. This ansatz establishes existence and provides an accessible way to generate fields.

**Figure 3:**
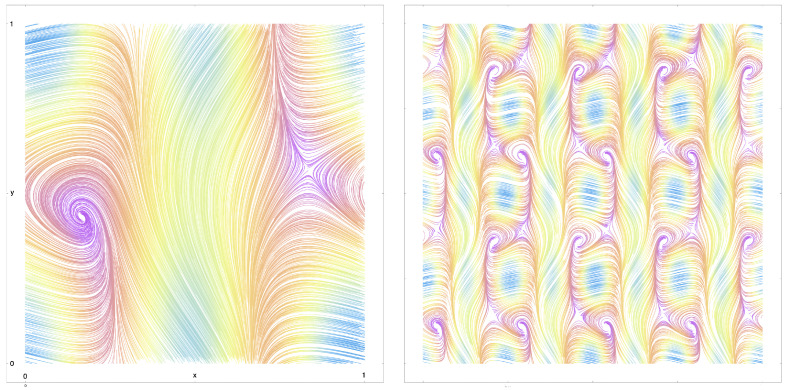
(**Left**): Streamlines for an example flow ${\left(X\left(x,y\right),Y\left(x,y\right)\right)}^{T}$ on K(1,1,(1)), the standard Klein bottle, with a saddle point and stable focus. (**Right**): The same flow showing the Klein bottle symmetries on an extended part of the plane, ${\left[0,4\right]}^{2}$: the flow is 1-periodic in the toroidal coordinate, *x*, and 2-periodic in the Klein coordinate, *y*, due to the flip symmetry. Streamlines coloured by norm of the vector field (light blue/fast through yellow then orange then purple/slow).

**Figure 4:**
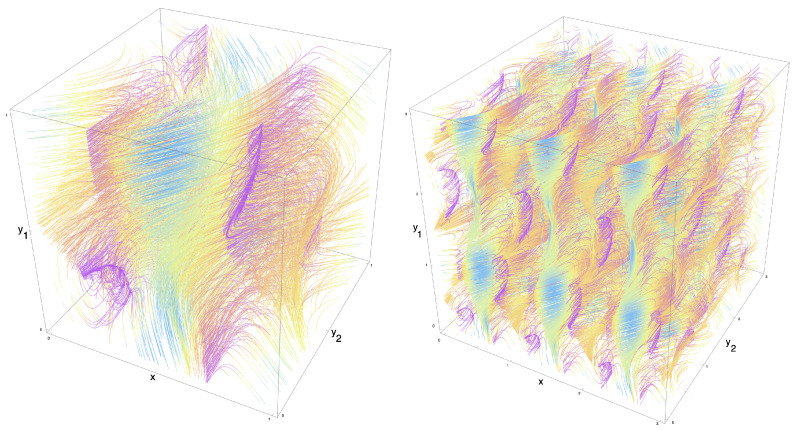
(**Left**): Streamlines for an example flow ${\left(X\left(x,y_{1},y_{2}\right),Y_{1}\left(x,y_{1},y_{2}\right),Y_{2}\left(x,y_{1},y_{2}\right)\right)}^{T}$ on K(1,2,(1,1)), with one toroidal and two Klein coordinates, containing a vortex filament. (**Right**): the same flow showing the K(1,2,(1,1)) symmetries on an extended volume, ${\left[0,3\right]}^{3}$: The flow is 1-periodic in the toroidal coordinate, *x*, and 2-period in both Klein coordinates, $y_{1}$ and $y_{2}$, due to the flip symmetries. Streamlines coloured by norm of the vector field (light blue/fast, through yellow then orange then purple/slow).

In Figure 3 and Figure 4, we depict the streamlines for two example flows that were generated in this manner, on K(1,1,(1)) and K(1,2,(1,1)), respectively.

**Figure 5:**
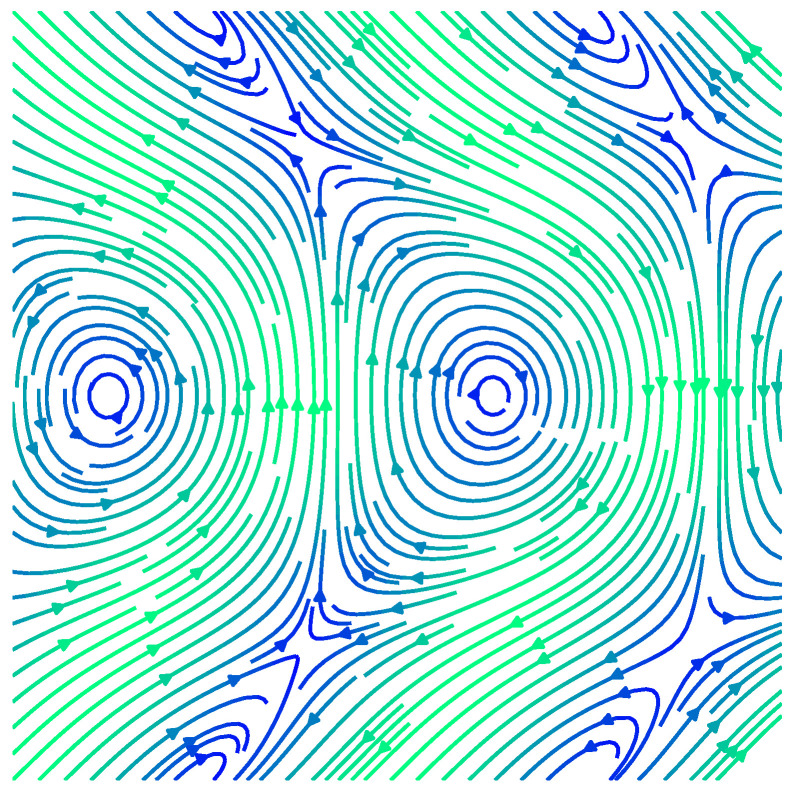
An example of a vector field (u,v) on the Klein bottle, illustrated here as a unit square domain where the top and bottom sides are identified with opposite orientation, and the left and right hand are identified with the same orientation. Here, u(x,y)=cos(2πx)cos(πy)+sin(2πx)sin(2πy), and v(x,y)=sin(2πx)sin(πy)+cos(2πx)cos(2πy). Note that vectors on the top and bottom edges of the square which are identified with opposite orientation have an extra flip in the *x*-coordinate of the vector field, reflecting the fact that the Klein bottle is not orientable. The colour scale indicates the magnitude of the vector field, increasing from blue (zero) to green.

In Appendix B, we find a Fourier basis for vector fields on $K(k_{1},k_{2},B)$. Similar to the approach in Appendix A, we first formulate the symmetry condition as being in the kernel of an operator on the vector space of vector fields. This correspondingly induces a constraint on the Fourier coefficients of the vector fields, expressed as being in the kernel of the dual of the aforementioned operator. We present an example of a vector field on the standard Klein bottle K(1,1) in Figure 5, constructed on a Fourier basis.

## 4. A Challenge: Spiking Dynamical Systems Modelling Neural Columns

Here, we discuss an application of dynamical systems with attractors set within compact manifolds having topological structures that need to be determined. This raises a number of challenges.

Human brains have evolved both architectures and dynamics to enable effective information processing with around $10^{10}$ neurons. These **spiking dynamical systems (SDSs)** represent challenges of large-scale state spaces containing many possible cyclic interactions between neurons. Such brains exhibit a directed *network-of-networks* architecture, with the *inner*, densely connected, networks of neurons called *neural columns* (see [3] and the references therein). Near-neighbouring neural columns have relatively sparse directed connections compared to the *outer* network (between pairs of neurons, one from each).

Following [4] (and subsequent very large-scale simulations [5]), we consider the dynamics of information processing within SDSs representing neural columns, with $10^{2}–10^{4}$ nodes representing the neurons. Each node is both *excitable* and *refractory*: if the node is in its *ready state* when it receives an incoming spike, from an upstream neighbour, then it instantaneously fires and emits an outgoing signal spike along the directed edges to each of its downstream neighbours, which takes a small time to arrive. Once it fires, there must be a short refractory period whilst the node recovers its local chemical equilibrium. During this time, it cannot re-fire and just ignores any further incoming spike signals. Once the refractory period is complete, the node returns to the *ready state*. The refractory time period prevents arbitrarily fast *bursting* (rapid repeat firing) phenomena.

**Figure 6:**
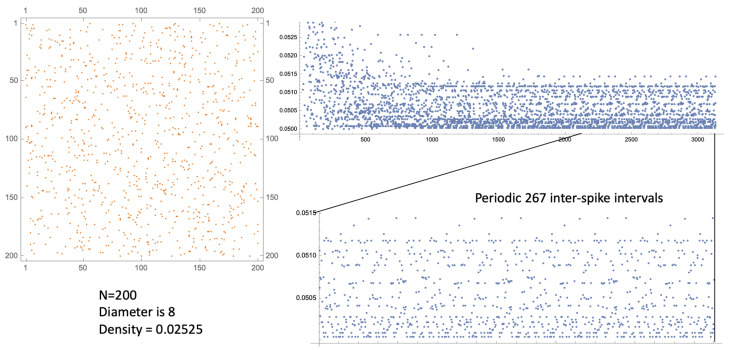
(**Left**): An adjacency matrix for a directed graph on N=200 nodes, with density ≈ 2.525% and network diameter 8. (**Right**): The successive inter-spike intervals (ISIs) at a single node. After a burn-in period of around 1500 spikes, the whole settles down to a long periodic orbit (with 267 successive ISIs) possibly containing many quasi-periodic features due to cycles within the network. Here, the edge transit times are independent and identically distributed random variables in U[0.5,1.5] and δ=0.050.

**Figure 7:**
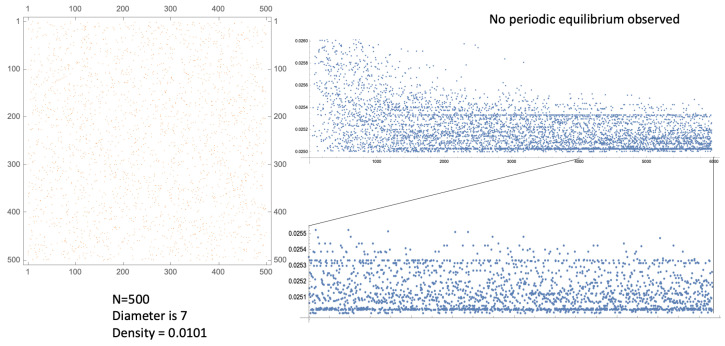
(**Left**): An adjacency matrix for a directed graph on N=500 nodes, with density ≈ 1.010% and network diameter 7. (**Right**): The successive inter-spike intervals (ISIs) at a single node, after a burn-in period of around 3500 spikes the whole settles down to quasi-periodic (possibly chaotic) behaviour, with no long period observed, containing many quasi-periodic features due to cycles within the network. The edge transit times are independent and identically distributed random variables in U[0.5,1.5] and δ=0.025.

We consider sparse directed networks that are irreducible, so that all nodes may be influenced by all others. The whole network should exhibit a relatively small diameter. We set an appropriate set of transit times for each directed edge, independently and identically drawn from a uniform distribution, and a common refractory period time, δ, for all nodes. Then, the whole SDS may be kick-started with a single spike at t=0 at a particular node, while all other nodes begin in their ready resting state. The dynamic results in a firing sequence for each node: a list of firing times at which that node instantaneously spikes. The SDS begins to chatter amongst itself and, after a burn-in period, it settles down to some very long-term pattern (see [6] for an observed instance). It is deterministic and possibly chaotic: see Figure 6 and Figure 7 for typical examples.

Since the network is irreducible, we cannot consider sub-networks of the nodes; and not all independent walks from one specific node to another may be viable: two such walks may result in spike arrival times less that δ apart, with the latter one ignored. However, the irredicibility does mean that in order to examine the dynamical behaviour of the whole system, it is enough to examine a single node. Furthermore, as the spikes are instantaneous, it is conventional within spike sequence analysis to examine the corresponding sequence of successive **inter-spike intervals (ISIs)** [6]. By definition, these are real and are bounded below by δ. The SDS model timescale is arbitrary: results depend only on the size of δ relative to the range of the independent and identically distributed random variable edge transit time.

The SDS examples in Figure 6 and Figure 7 illustrate these features. The ISI sequences suggest dynamics within a bounded attractor, that lies within some manifold, M, say, of unknown dimension and topology. Generalised Klein bottles and tori are candidates for M.

We embed the observed ISI sequence as a point cloud within $R^{L}$ by discarding the ISI burn-in and moving a window of length *L* successively along the sequence. Then, we estimate the dimension, $D_{M}\left(L\right)$, of the curved manifold, $M\subset R^{L}$, on which that point cloud lives, from the set of all pairwise distances between points within the cloud via the 2NN (two nearest neighbour) method [7]. For a small *L*, the point cloud (and M) merely fills up the available dimensions, so $D_{M}\left(L\right)∼L$. As *L* increases further, we have estimates $D_{M}\left(L\right)<<L$.

Considering a post burn-in ISI sequence of length 1900 from the example given in Figure 7, we need to take L≥40 to avoid any duplicate points from windows along the sequence (which we know to be non-periodic). For lower values of *L*, we remove any duplicates from the point cloud (which otherwise interfere with the 2NN algorithm).

**Figure 8:**
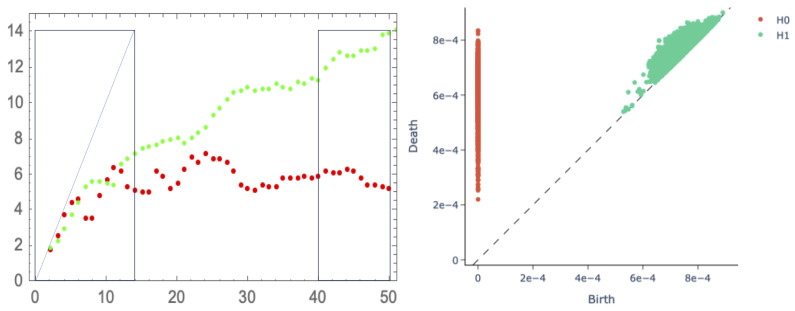
(**Left**): Plot of $D_{M}\left(L\right)$ versus *L* using the 2NN method applied to the moving windows of length *L*, taken from a post burn-in ISI sequence. Green: from a sequence of 1900 points from the non-periodic SDS given in Figure 7. Red: from a sequence of 267 points from the 267-periodic SDS given in Figure 6. (**Right**): A persistent homology analysis for the point cloud representing the (post-burn-in) 267-periodic SDS given in Figure 6, for L=16, using the Rips filtration (based on pairwise distances between the points). Red points show the ($H_{0}$) features of connected components as the filtration scale increases, while green points show the ($H_{1}$) features corresponding to distinct rings around holes through the cloud: such features close to the diagonal are non-persistent and represent sampling noise.

We apply this method to the post burn-in ISI sequence, of length 1900, given in Figure 7 in Figure 8 (Left, Green). This suggests dimension, $D_{M}$, of 5 to 7 at the first obvious plateau, embedded in L=7 to 11 dimensions. If *L* is increased further, then $D_{M}\left(L\right)$ increases rather slowly as the attractor *fills in* somewhat. For comparison, we show the result for the 267-periodic case given in Figure 6 (left, red) where, necessarily, we have 267 windowed points. This suggests a dimension, $D_{M}$, between four and six.

It is clear that this class of SDSs gives rise to highish dimensional compact attractors. What is required is a pipeline that begins from a suitable embedding of the post-burn-in ISIs into some $R^{L}$ (just as above, which determined the dimension, $D_{M}\left(L\right)$, of the manifold, M, containing the attractor), followed by a computational method (most likely based on persistent homology) that can determine (a) the main topological features of the manifold containing the point cloud, and subsequently (much more challenging) (b) the topological symmetries that can differentiate between various generalised Klein bottles of given dimensions ($k_{1}+k_{2}\approx D_{M}\left(L\right)$).

Following Figure 8 (left) and embedding the 267-periodic ISI from Figure 6 into L=16 dimensions (whence the estimate $D_{M}\left(L\right)\approx 5.1$), a persistent homology (PH) analysis is shown in Figure 8 (right). It is somewhat inconclusive, possibly due to the variations in the localised cloud density. This issue is the subject of active research.

It is this characterisation of the SDS’s unknown attracting manifolds that drives the permissive generalisation of Klein bottles presented in this paper. This is the subject of active ongoing research.

## 5. Discussion

In this paper, we introduced a generalisation of the standard Klein bottle to higher dimensions, beyond those previously considered. We focused on a subclass where the automorphisms are *diagonal*, inducing flips (reflections) of some independent coordinates.

For all such Klein bottles, we produced both a simple (practical) method and a rigorous argument for the definition of scalar fields (such as smooth probability distributions and potentials) as well as vector fields (flows) that are well defined. These constructions are useful when we wish to consider winding flows over Klein bottles and possibly couple them together.

This class of Klein bottles can be extended to include automorphisms that induce swaps (permutations) between toroidal coordinates (diagonal reflections rather than individual coordinate reflections). In future work, we will extend this approach to consider an even wider class of compact manifolds without boundaries, including real projective geometries, for which there is not a partition of coordinates into Klein variables (controlling the automorphisms), and toroidal variables (acted on by the automorphisms). Since spheres are the universal covering spaces of projective spaces, we would consider lifts of vector fields on the projective plane to its universal covering sphere to parametrise them and express their Fourier basis with spherical harmonics that respect the required symmetries.

Finally, we set out a challenging application where high-dimensional spiking dynamical systems result in attractors over unknown compact manifolds which require characterisation. This is highly problematic when the dimension of such spiking systems is very large and the resulting ISI sequences have to be embedded within a suitable space. The identification of the dimension and the topological properties of resultant attractor manifolds is both a theoretical and a computational challenge.

## Figures and Tables

**Figure A1:**
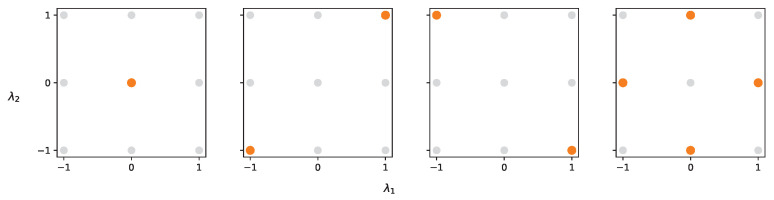
The orbits (labelled orange) of the action of automorphisms of $Z^{2}$, parametrised by Equation (A14), on $\left(\lambda_{1},\lambda_{2}\right)\in {\left\{-1,0,1\right\}}^{2}$.

## Data Availability

The data presented in this study are available on request from the corresponding author.

## Appendix A. Fourier Modes of Scalar Fields on Klein Bottles

We recall the Klein group action on $R^{k_{1}}\times R^{k_{2}}$ described above. Given homomorphism $\varphi :Z^{k_{2}}\to \mathrm{Aut}\left(Z^{k_{1}}\right)$, we have the action of group $G=Z^{k_{1}}{⋊}_{\varphi }Z^{k_{2}}$ on $R^{k_{1}}\times R^{k_{2}}$, where for $\lambda \in Z^{k_{1}}$ and $\zeta \in Z^{k_{2}}$,

(A1) $$\begin{array}{c} (\lambda ,\zeta )\cdot (x,y)=(\varphi (\zeta ).x+\lambda ,y+\zeta ). \end{array}$$

Note that the action of *G* on $R^{n}$ is a covering space action—for any element not equal to identity (0,0), the action shifts at least one coordinate by an integer, so there is a sufficiently small neighbourhood *U* of any point such that g·U∩U= for g≠(0,0).

If $\varphi {\left(\zeta \right)}^{2}=1$ for all $\zeta \in Z^{k_{2}}$, then $H=Z^{k_{1}}\times {\left(2Z\right)}^{k_{2}}$ is a normal subgroup of $G=Z^{k_{1}}{⋊}_{\varphi }Z^{k_{2}}$. Thus, we can factor the quotient of $R^{n}$ by *G* into two successive quotients:

(A2) $$\begin{array}{c} R^{n}\to R^{n}/H\to R^{n}/G. \end{array}$$

The first quotient is the quotient of $R^{n}$ by the action of subgroup $H=Z^{k_{1}}\times {\left(2Z\right)}^{k_{2}}$ on $R^{n}$, where due to $\varphi \left(2\zeta \right)=\varphi {\left(\zeta \right)}^{2}=1$,

(A3) (λ,2ζ)·(x,y)=(x+λ,y+2ζ).

Thus, $R^{n}/H$ is diffeomorphic to the *n*-torus T where the aspect ratio of any of the first $k_{1}$ coordinates to the last $k_{2}$ coordinates is 1:2. Because the action of *G* on $R^{n}$ is a covering space action and *H* is a normal subgroup, the quotient $q:T=R^{n}/H\to R^{n}/G$ is a covering obtained by the quotient of T by a covering space action of group $G/H≅Z_{2}^{k_{2}}$ on T. Writing elements of T as $z=(e^{2\pi ix_{1}},\ldots ,e^{2\pi ix_{k_{1}}},e^{\pi iy_{1}},\ldots ,e^{\pi iy_{k_{2}}})$, the action of element $\beta =\left(\beta_{1},\ldots ,\beta_{k_{2}}\right)\in Z_{2}^{k_{2}}$ on such an element is given by

(A4) $$\begin{array}{cc} & \beta \cdot (e^{2\pi ix_{1}},\ldots ,e^{2\pi ix_{k_{1}}},e^{\pi iy_{1}},\ldots ,e^{\pi iy_{k_{2}}})\\ & =(e^{2\pi i{\left(\varphi \left(\beta \right)x\right)}_{1}},\ldots ,e^{2\pi i{\left(\varphi \left(\beta \right)x\right)}_{k_{1}}},e^{\pi i(y_{1}+\beta_{1})},\ldots ,e^{\pi i(y_{k_{2}}+\beta_{k_{2}})}). \end{array}$$

If we consider functions f:K→C, they lift to a function on the torus $q^{*}f:T\to C$, defined by function $q^{*}f=f\circ q$ sending elements of T to *K* by *q* and evaluating *f* on the image of those points. The lifted function $q^{*}f$ is called the *pull back* of *f*. Since *q* is surjective, $q^{*}$ is an injection of C-valued functions on *K* into those of T. Function h:T→C is the lift of some function on *K*, if for every y∈K, function *h* is constant on $f^{-1}\left(y\right)$, that is, the orbit of a representative $z\in f^{-1}\left(y\right)$

(A5) h(z)=h(g·z)   ∀z∈T, g∈G.

We invoke Lemma A2, which implies *h* satisfies the symmetry above if it is in the kernel of a *linear* operator L:C[T]→C[T] on the vector space of C[T] of complex valued functions on T, where

(A6) $$\left(Lh\right)\left(z\right)=h\left(z\right)-\frac{1}{2^{k_{2}}}\sum_{\beta \in Z_{2}}h\left(\beta \cdot z\right).$$

If *h* admits a Fourier transform, then *h* satisfies the symmetry if the Fourier coefficients $c\in C[Z^{k_{1}}\times Z^{k_{2}}]$ of *h* are in the kernel of linear operator

(A7) $$\left(L^{★}c\right)\left(\lambda ,\zeta \right)=c\left(\lambda ,\zeta \right)-\frac{1}{2^{k_{2}}}\sum_{\beta \in Z_{2}^{k_{2}}}{\left(-1\right)}^{\zeta .\beta }c\left(\varphi {\left(\beta \right)}^{T}\lambda ,\zeta \right).$$

This is a consequence of Lemma A1. We further remark that while $L^{★}$ is an operator on an infinite dimensional space, it can be expressed as a direct sum of countably many finite dimensional operators (Remark A2).

We offer some examples below.

**Example** **A1** (The standard Klein bottle). *The standard Klein bottle has $k_{1}=k_{2}=1$. The induced homomorphism $\varphi :Z_{2}\to \mathrm{Aut}\left(Z\right)$ is given by $\varphi \left(\beta \right)={\left(-1\right)}^{\beta }$. The dual operator $L^{★}:C\left[Z\times Z\right]\to C\left[Z\times Z\right]$, Equation (A7), is given by*

(A8) $$\begin{array}{cc} L^{★}c\left(\lambda ,\zeta \right) & =c\left(\lambda ,\zeta \right)-\frac{1}{2}\sum_{\beta \in Z_{2}}{\left(-1\right)}^{\zeta .\beta }c\left(\varphi \left(\beta \right)\lambda ,\zeta \right)\\ & =\frac{1}{2}\left(c\left(\lambda ,\zeta \right)-{\left(-1\right)}^{\zeta }c\left(-\lambda ,\zeta \right)\right) \end{array}$$

*The kernel is given by*

(A9) $$\begin{array}{c} c\in \ker L^{★}\Leftrightarrow \left\{\begin{array}{cc} c(\lambda ,\zeta )=c(-\lambda ,\zeta ) & \zeta \in 2Z\\ c(\lambda ,\zeta )=-c(-\lambda ,\zeta ) & \zeta \in 2Z+1. \end{array}\right. \end{array}$$

*In other words, $\ker L^{★}$ admits a basis of indicator functions on Z×Z,*

(A10) $$\begin{array}{c} \ker L^{★}=\mathrm{span}\left(\left\{\begin{array}{cc} 1_{\left(\lambda ,\zeta \right)}+1_{\left(-\lambda ,\zeta \right)} & \lambda \in Z_{\geq 0},\;\zeta \in 2Z\\ 1_{\left(\lambda ,\zeta \right)}-1_{\left(-\lambda ,\zeta \right)} & \lambda \in Z_{\geq 0},\;\zeta \in 2Z+1 \end{array}\right.\right). \end{array}$$

*Taking the inverse Fourier transform of these basis functions, we obtain the Fourier basis of functions lifted from the Klein bottle to the torus,*

(A11) $$\begin{array}{c} \ker L=\mathrm{span}\left(\left\{\begin{array}{cc} \cos\left(2\pi \lambda x\right)e^{\pi i\zeta y} & \lambda \in Z_{\geq 0},\;\zeta \in 2Z\\ \sin\left(2\pi \lambda x\right)e^{\pi i\zeta y} & \lambda \in Z_{\geq 0},\;\zeta \in 2Z+1 \end{array}\right.\right). \end{array}$$

*We can verify that these basis functions are invariant under action $\left(\lambda ,\zeta \right)\cdot \left(x,y\right)=({\left(-1\right)}^{\zeta }x+\lambda ,y+\zeta )$.*

**Example** **A2** (Transpose and Flip). *We take $k_{1}=k_{2}=2$, and set $\varphi :Z^{2}\to \mathrm{Aut}\left(Z^{2}\right)$ to be the following homomorphism expressed in matrix form as*

(A12) $$\varphi \left(n_{1},n_{2}\right)={\left(-1\right)}^{n_{1}}{\left(\begin{array}{cc} 0 & 1\\ 1 & 0 \end{array}\right)}^{n_{2}}.$$

*We let $\left(\lambda_{1},\lambda_{2},\zeta_{1},\zeta_{2}\right)\in Z^{k_{1}}\times Z^{k_{2}}$ denote the Fourier variables. Let us consider Fourier coefficients with $\left(\zeta_{1},\zeta_{2}\right)=\left(1,1\right)$. We also restrict to the subspace of coefficients where $\left(\lambda_{1},\lambda_{2}\right)\in {\left\{-1,0,1\right\}}^{2}$. Setting v(λ)=c(λ,(1,1)) as a shorthand, the dual operator $L^{★}$ acts on the the vector of coefficients by the following matrix:*

(A13) L*||λi|≤1, ζ=(1,1)v=144     4     4     22    22     31−11   131−1   −1131   1−113v(0,0)v(−1,−1)v(1,1)v(−1,1)v(1,−1)v(−1,0)v(0,−1)v(0,1)v(1,0).

*The blocks correspond to λ’s in $Z^{2}$ on the same orbit of the the action of automorphisms on $\left(\lambda_{1},\lambda_{2}\right)\in Z^{2}$:*

(A14) $$\beta \cdot \lambda =\varphi {\left(\beta_{1},\beta_{2}\right)}^{T}\lambda ={\left(-1\right)}^{\beta_{1}}{\left(\begin{array}{cc} 0 & 1\\ 1 & 0 \end{array}\right)}^{\beta_{2}}\left(\begin{array}{c} \lambda_{1}\\ \lambda_{2} \end{array}\right).$$

*The orbits are illustrated in Figure A1.*

*We can then compute the kernel of $L^{★}$ on the subspace of coefficients, which is two-dimensional in a subspace of Dimension 9, and admits an orthogonal basis*

(A15)
$$\begin{array}{c} \ker L^{*}{|}_{|\lambda_{i}|\leq 1,\;\zeta }\\ =\{v(-1,1)=v(1,-1),\;-v(-1,0)+v(0,-1)-v(0,1)+v(1,0)=0\} \end{array}$$

*Taking the inverse Fourier transform of the basis vectors above, we obtain a basis for the kernel of L restricted to these Fourier modes $|\lambda_{i}|\leq 1,\zeta =\left(1,1\right)$:*

(A16)
$$F^{-1}{\left(\ker L^{*}\right|}_{|\lambda_{i}|\leq 1,\;\zeta })=\mathrm{span}\{\sin\left(2\pi \left(x_{1}-x_{2}\right)\right)e^{\pi i(y_{1}+y_{2})},$$

(A17)
$$\left(\sin\left(2\pi x_{1}\right)-\sin\left(2\pi x_{2}\right)\right)e^{\pi i(y_{1}+y_{2})}\}\subset \ker L.$$

*We can then check that these are a basis for the functions that are invariant under the automorphisms φ(β) restricted to the Fourier modes above.*

**Example** **A3** (Double Flip). *We consider the generalised Klein bottle K(2,2,B), where B Set $\varphi :Z^{2}\to \mathrm{Aut}\left(Z^{2}\right)$, to be the following homomorphism expressed in matrix form as*

(A18) $$\varphi \left(n_{1},n_{2}\right)=\left(\begin{array}{cc} {\left(-1\right)}^{n_{1}} & 0\\ 0 & {\left(-1\right)}^{n_{2}} \end{array}\right).$$

*As with the example above, let us restrict to Fourier modes with $\left(\zeta_{1},\zeta_{2}\right)=\left(1,1\right)$, and $\left(\lambda_{1},\lambda_{2}\right)\in {\left\{-1,0,1\right\}}^{2}$. Repeating the same procedure, we obtain a basis for the kernel of L restricted to these Fourier modes:*

(A19) $$\begin{array}{c} \mathrm{span}\{\sin\left(2\pi x_{1}\right)\sin\left(2\pi x_{2}\right)e^{i\pi (y_{1}+y_{2})}\}\subset \ker L. \end{array}$$

*We can then check that these are a basis for the functions that are invariant under the automorphisms φ(β) restricted to the Fourier modes above.*

## Appendix B. Fourier Modes of Vector Fields on Klein Bottles

We now apply the same treatment to vector fields. We first consider local coordinate transformations along orbits of the group action on T. Note that the tangent bundle of the torus is simply a trivial bundle $TT≅T\times R^{n}$, and vectors can be parallel translated around T as in Euclidean space since we are working with the flat torus. This allows us to fix a global Euclidean coordinate frame across all of the tangent spaces $T_{p}T$ in the tangent bundle TT and write vector fields as function $V:T\to R^{n}$.

We now consider how our $Z_{2}^{k_{2}}$-group action on T induces a group action on TT. We recall that diffeomorphisms between smooth manifolds induce diffeomorphisms between their respective tangent bundles. We recall the $Z_{2}^{k_{2}}$-action is a subgroup of the group of diffeomorphism of T. Action z↦β·z induces a transformation that sends vector $v\in T_{z}T$ to vector $\beta \cdot v\in T_{\beta \cdot z}T$. Since vectors transform via matrix multiplication by the Jacobian, and $\varphi \left(\beta \right)⊕1_{k_{2}}$ is the Jacobian of the transformation, we have

(A20) $$\begin{array}{c} \beta \cdot v=\underset{A(\beta )}{\underset{︸}{\left(\begin{array}{cc} \varphi (\beta ) & 0\\ 0 & 1_{k_{2}} \end{array}\right)}}v \end{array}$$

Note that A(β)∈GL(n,Z). Vector field *V* on the torus is invariant with respect to our group action, if for all $\beta \in Z_{2}^{k_{2}}$,

(A21) V(β·z)=A(β)V(z).

We can express any vector field on K as a vector field on T satisfying Equation (A21), and any vector field on T satisfying Equation (A21) represents a vector field on K. We recall q:T→K is a local diffeomorphism. For z∈T and q(z)∈K, we can choose a sufficiently small neighbourhood *U* of *z* such that $q^{-1}\left(q\left(U\right)\right)={⊔}_{\beta }\beta \cdot U$. Because *q* is a smooth covering, the restriction ${q|}_{U}:U\to q\left(U\right)$ is a diffeomorphism, and induces isomorphisms of vector fields defined on *U* and q(U). Because the group action sends *U* diffeomorphically to all other disjoint copies in $q^{-1}\left(q\left(U\right)\right)$, any vector field defined on q(U) pulls back to a vector field on $q^{-1}\left(q\left(U\right)\right)$, satisfying Equation (A21); conversely, any vector field on *U* satisfying Equation (A21) pushes forward to a well-defined vector field on q(U). Because this holds on every element of a sufficiently fine open cover of K, we deduce that any vector field *W* on K pulls back to a vector field $q^{*}W$ on T, satisfying Equation (A21), and any vector field *V* on T satisfying Equation (A21) pushes forward to a vector field $q_{*}V$ on K; we also note that if *W* can be pulled back, then $q_{*}q^{*}W=W$; and similarly, if *V* can be pushed forward, then $q^{*}q_{*}V=V$.

Applying Lemma A2, vector field $V:T\to R^{n}$ satisfies Equation (A21) if it is in the kernel of operator

(A22) $$\left(LV\right)\left(z\right)=V\left(z\right)-\frac{1}{2^{k_{2}}}\sum_{\beta \in Z_{2}^{k_{2}}}A\left(\beta \right)V\left(\beta \cdot z\right).$$

Writing V=(X,Y) where $X:T\to R^{k_{1}}$ and $Y:T\to R^{k_{2}}$, condition V∈kerL can be rewritten as follows, since $A\left(\beta \right)=\varphi \left(\beta \right)⊕1_{k_{2}}$ is block diagonal:

(A23) $$\begin{array}{cc} X(z) & =\frac{1}{2^{k_{2}}}\sum_{\beta \in Z_{2}^{k_{2}}}\varphi \left(\beta \right)X\left(\beta \cdot z\right) \end{array}$$

(A24) $$\begin{array}{cc} Y(z) & =\frac{1}{2^{k_{2}}}\sum_{\beta \in Z_{2}^{k_{2}}}Y\left(\beta \cdot z\right). \end{array}$$

Note that the condition on each *Y* coordinate is simply the scalar field condition. We focus on the *X* coordinates of the vector field. If *X* admits a Fourier transform, then Lemma A1 implies the Fourier coefficients $c=F\left(X\right):Z^{k_{1}}\times Z^{k_{2}}\to C^{k_{1}}$ are given by the kernel of the following operator:

(A25) $$\begin{array}{c} \left(L_{X}^{★}c\right)\left(\lambda ,\zeta \right)=c\left(\lambda ,\zeta \right)-\frac{1}{2^{k_{2}}}\sum_{\beta \in Z_{2}^{k_{2}}}{\left(-1\right)}^{\zeta .\beta }\varphi \left(\beta \right)c\left(\varphi {\left(\beta \right)}^{T}\lambda ,\zeta \right). \end{array}$$

**Example** **A4** (The standard Klein bottle). *For the Klein bottle K(1,1), the operator $L_{X}^{★}$ (Equation (A25)) on the Fourier coefficient c:Z→C of the X component of the vector field V is*

(A26) $$\begin{array}{c} \left(L_{X}^{★}c\right)\left(\lambda ,\zeta \right)=\frac{1}{2}\left(c(\lambda ,\zeta )+c(-\lambda ,\zeta )\right). \end{array}$$

*By solving for the kernel of $L_{X}^{★}$, the X component admits a Fourier basis,*

(A27) $$\begin{array}{cc} X\in \mathrm{span}( & \left\{\sin\left(2\pi \lambda x\right)e^{\pi i\zeta y}\;|\;\lambda \in Z_{\geq 0},\;\zeta \in 2Z\right\}\\ & \cup \;\{\cos\left(2\pi \lambda x\right)e^{\pi i\zeta y}\;|\;\lambda \in Z_{\geq 0},\;\zeta \in 2Z+1\}). \end{array}$$

*Because the Y coordinate satisfies the same constraints as the scalar field case,*

(A28) $$\begin{array}{cc} Y\in \mathrm{span}( & \left\{\cos\left(2\pi \lambda x\right)e^{\pi i\zeta y}\;|\;\lambda \in Z_{\geq 0},\;\zeta \in 2Z\right\}\\ & \cup \;\{\sin\left(2\pi \lambda x\right)e^{\pi i\zeta y}\;|\;\lambda \in Z_{\geq 0},\;\zeta \in 2Z+1\}). \end{array}$$

*Thus, vector fields on T with the following Fourier series pushes forward to a vector field on the Klein bottle K:*

(A29) $$\begin{array}{c} V\left(e^{2\pi ix},e^{\pi iy}\right)=\sum_{\lambda \geq 0}\sum_{\zeta \in 2Z}\left(\begin{array}{c} r_{\lambda ,\zeta }\sin\left(2\pi \lambda x\right)\\ s_{\lambda ,\zeta }\cos\left(2\pi \lambda x\right) \end{array}\right)e^{i\pi \zeta y}+\sum_{\zeta \in 2Z+1}\left(\begin{array}{c} r_{\lambda ,\zeta }\cos\left(2\pi \lambda x\right)\\ s_{\lambda ,\zeta }\sin\left(2\pi \lambda x\right) \end{array}\right)e^{i\pi \zeta y} \end{array}$$

## Appendix C. Technical Lemmas for Group Actions and Symmetries

We consider the group of affine transformations $\mathrm{Aff}(R^{n})$ on $R^{n}$. For $b\in R^{n}$ and A∈GL(n,R) an invertible matrix, we have an affine action on $R^{n}$ given by

(A30) (b,A)·x=A.x+b

The affine group is a semi-direct product $\mathrm{Aff}\left(R^{n}\right)=R^{n}⋊\mathrm{GL}\left(n,R\right)$, where

(A31) $$\left(b^{\prime },A^{\prime }\right)*\left(b,A\right)=\left(b^{\prime }+A^{\prime }b,A^{\prime }A\right).$$

We can check that this is the choice of product structure such that it is compatible with the composition of affine transformations, satisfying

(A32) $$\left(b^{\prime },A^{\prime }\right)\cdot \left(\left(b,A\right)\cdot x\right)=\left(\left(b^{\prime },A^{\prime }\right)*\left(b,A\right)\right)\cdot x.$$

Suppose we restrict to a subgroup of affine transformations, parametrised over another group *G*. In other words, we have a group homomorphism $\Phi :G\to \mathrm{Aff}(R^{n})$, which we write as

(A33) Φ: g↦(b(g),A(g))

where A:G→GL(n,R) and $b:G\to R^{n}$ are functions. The group homomorphism condition on Φ enforces that *A* is a group homomorphism. As for *b*, since

(A34) b(g·h)=A(g)b(h)+b(g),

unless *A* maps every element of *G* to the identity matrix, *b* is notably not a homomorphism.

**Remark** **A1.**
*In the case of the generalised Klein bottle, we consider $G=Z^{k_{1}}{⋊}_{\varphi }Z^{k_{2}}$ to be the semi-direct product between $Z^{k_{1}}$ and $Z^{k_{2}}$, specified by $\varphi :Z^{k_{2}}\to \mathrm{Aut}\left(Z^{k_{1}}\right)$. Writing an element of G as (λ,ζ) where $\lambda \in Z^{k_{1}}$, and $\zeta \in Z^{k_{2}}$, we set $b=(b_{1},b_{2})$ where $b_{1}\left(\lambda ,\zeta \right)=\lambda$ and $b_{2}\left(\lambda ,\zeta \right)=\zeta$ simply embeds the integer tuple as real coordinates. Thus,*

(A35)
$$b_{1}\left(\left(\lambda^{\prime },\zeta^{\prime }\right)\cdot \left(\lambda ,\zeta \right)\right)=A_{11}\left(\lambda^{\prime },\zeta^{\prime }\right)b_{1}\left(\lambda ,\zeta \right)+A_{12}\left(\lambda^{\prime },\zeta^{\prime }\right)b_{2}\left(\lambda ,\zeta \right)+b_{1}\left(\lambda^{\prime },\zeta^{\prime }\right)$$

(A36)
$$\Rightarrow \varphi (\zeta^{\prime })\lambda =A_{11}\left(\lambda^{\prime },\zeta^{\prime }\right)\lambda +A_{12}\left(\lambda^{\prime },\zeta^{\prime }\right)\zeta$$

(A37)
$$b_{2}\left(\left(\lambda^{\prime },\zeta^{\prime }\right)\cdot \left(\lambda ,\zeta \right)\right)=A_{21}\left(\lambda^{\prime },\zeta^{\prime }\right)b_{1}\left(\lambda ,\zeta \right)+A_{22}\left(\lambda^{\prime },\zeta^{\prime }\right)b_{2}\left(\lambda ,\zeta \right)+b_{2}\left(\lambda^{\prime },\zeta^{\prime }\right)$$

(A38)
$$\Rightarrow \zeta =A_{21}\left(\lambda^{\prime },\zeta^{\prime }\right)\lambda +A_{22}\left(\lambda^{\prime },\zeta^{\prime }\right)\zeta .$$

*Since these relations hold for arbitrary (λ,ζ), we conclude that $A_{11}=\varphi$, $A_{12}=A_{21}=0$. Thus,*

(A39)
(λ,ζ)·(x,y)=(φ(ζ).x+λ,y+ζ).

**Lemma** **A1.**
*We let T be an n-torus equipped with a G-action, where G is a finite group and acts on $z=(e^{2\pi i\theta_{1}},\ldots ,e^{2\pi i\theta_{n}})\in T$ by transformation*

(A40)
$$g\cdot z=(e^{2\pi i{\left(A\left(g\right)\theta +b\left(g\right)\right)}_{1}},\ldots ,e^{2\pi i{\left(A\left(g\right)\theta +b\left(g\right)\right)}_{n}})$$

*where A:G→GL(n,Z) is a homomorphism, and b(g) satisfies*

b(g·h)=A(g)b(h)+b(g) mod 1

*so that the above transformation is a group action. We consider the dual operator $L^{★}:C^{n}\left[Z^{n}\right]\to C^{n}\left[Z^{n}\right]$ of $L:C^{n}\left[T\right]\to C^{n}\left[T\right]$ in Equation (A44), defined via*

(A41)
$$L^{★}=FLF^{-1}.$$

*Then, $L^{★}$ is explicitly given on $c\in C[Z^{n}]$, $k\in Z^{n}$ by*

(A42)
$$\left(L^{★}c\right)\left(k\right)=c\left(k\right)-\frac{1}{\left|G\right|}\sum_{g\in G}\chi \left(g^{-1}\right)c\left(A{\left(g^{-1}\right)}^{T}k\right)e^{2\pi ik^{T}A\left(g^{-1}\right)b\left(g\right)}.$$

**Proof.** We consider $c\in C^{n}\left[Z^{n}\right]$.

$$\begin{array}{cc} L^{★}c & =FLF^{-1}c=F\left(L\int c\left(k\right)e^{2\pi ik}dk\right)=F\left(\int L\left(c\left(k\right)e^{2\pi ik}\right)dk\right), \end{array}$$

where we have the last equality due to linearity. Thus,

$$\begin{array}{cc} L\left(c\left(k\right)e^{2\pi ik}\right) & =c\left(k\right)e^{2\pi ik}-\frac{1}{\left|G\right|}\sum_{g\in G}\chi \left(g^{-1}\right)c\left(k\right)e^{2\pi ik}\\ \Rightarrow \int L\left(c\left(k\right)e^{2\pi ik}\right)dk & =F^{-1}\left(c\right)-\frac{1}{\left|G\right|}\sum_{g\in G}\chi \left(g^{-1}\right)\int c\left(k\right)e^{2\pi ik^{T}\left(A\left(g\right)x+b\left(g\right)\right)}dk\\ & =F^{-1}\left(c\right)-\frac{1}{\left|G\right|}\sum_{g\in G}\chi \left(g^{-1}\right)\int c\left(A{\left(g^{-1}\right)}^{T}q\right)e^{2\pi iq^{T}A\left(g^{-1}\right)b\left(g\right)}e^{2\pi iq^{T}x}dq\\ & =F^{-1}\left(c\right)-\frac{1}{\left|G\right|}\int \sum_{g\in G}\chi \left(g^{-1}\right)c\left(A{\left(g^{-1}\right)}^{T}q\right)e^{2\pi iq^{T}A\left(g^{-1}\right)b\left(g\right)}e^{2\pi iq^{T}x}dq\\ & =F^{-1}\left(c\left(q\right)-\frac{1}{\left|G\right|}\sum_{g\in G}\chi \left(g^{-1}\right)c\left(A{\left(g^{-1}\right)}^{T}q\right)e^{2\pi iq^{T}A\left(g^{-1}\right)b\left(g\right)}\right) \end{array}$$

Applying the F on both sides,

$$\begin{array}{cc} \left(L^{★}c\right)\left(q\right) & =F\left(\int L\left(c\left(k\right)e^{2\pi ik}\right)dk\right)\left(q\right)\\ & =c\left(q\right)-\frac{1}{\left|G\right|}\sum_{g\in G}\chi \left(g^{-1}\right)c\left(A{\left(g^{-1}\right)}^{T}q\right)e^{2\pi iq^{T}A\left(g^{-1}\right)b\left(g\right)}. \end{array}$$

□

**Remark** **A2.**
*Operator $L^{★}$ in Equation (A42) is block diagonal: partitioning $Z^{n}$ into the space of orbits $Z^{n}/G$ under the induced group action on the dual Fourier space,*

(A43)
$$g\cdot k=A{\left(g^{-1}\right)}^{T}k,$$

*and writing $C^{n}\left[Z^{n}\right]$ as a direct sum over the finite dimensional subspaces $C^{n}\left[Z^{n}\right]={⨁}_{\left[k\right]\in Z^{n}/G}C^{n}\left[\left[k\right]\right]$, operator $L^{★}$ can be written as a direct sum of finite dimensional matrices on each subspace $C^{n}\left[\left[k\right]\right]$.*

**Lemma** **A2.**
*We let X be a set equipped with a G-action, where G is a finite group. We also consider χ:G→GL(n,C) to be a group homomorphism, such that χ(g∗h)=χ(g)χ(h). Then, function $f:X\to C^{n}$ satisfies f(g·x)=χ(g)f(x) for all g∈G, if it is in the kernel of the following linear operator L on $C^{n}$-valued functions on X:*

(A44)
$$\left(Lf\right)\left(x\right)=f\left(x\right)-\frac{1}{\left|G\right|}\sum_{g\in G}\chi \left(g^{-1}\right)f\left(g\cdot x\right).$$

**Proof.** We suppose f(g·x)=χ(g)f(x). Then,

$$\begin{array}{c} \sum_{g\in G}\chi \left(g^{-1}\right)f\left(g\cdot x\right)=\sum_{g\in G}\chi \left(g^{-1}\right)\chi \left(g\right)f\left(x\right)=\sum_{g\in G}\chi \left(g^{-1}g\right)f\left(x\right)=\sum_{g\in G}f\left(x\right)=\left|G\right|f\left(x\right). \end{array}$$

Thus, $\left(Lf\right)\left(x\right)=f\left(x\right)-\frac{1}{\left|G\right|}\sum_{g\in G}\chi \left(g^{-1}\right)f\left(g\cdot x\right)=0$, i.e., f∈kerL. Conversely, if f∈kerL, then for all h∈G,

$$\begin{array}{cc} f(h\cdot x) & =\frac{1}{\left|G\right|}\sum_{g\in G}\chi \left(g^{-1}\right)f\left(g\cdot h\cdot x\right)\\ & =\frac{1}{\left|G\right|}\sum_{g^{\prime }\in G}\chi \left({\left(g^{\prime }h^{-1}\right)}^{-1}\right)f\left(g^{\prime }\cdot x\right)\\ & =\frac{1}{\left|G\right|}\sum_{g^{\prime }\in G}\chi \left(h\right)\chi \left({g^{\prime }}^{-1}\right)f\left(g^{\prime }\cdot x\right)\\ & =\chi \left(h\right)\left(\frac{1}{\left|G\right|}\sum_{g^{\prime }\in G}\chi \left({g^{\prime }}^{-1}\right)f\left(g^{\prime }\cdot x\right)\right)=\chi \left(h\right)f\left(x\right). \end{array}$$

□

**Remark** **A3.**
*If χ(g)=1, then operator L in Equation (A44) can be interpreted as a graph Laplacian. For each orbit $x_{1},\ldots ,x_{n}$, we construct a multigraph where the vertex set consists of the $x_{1},\ldots ,x_{n}$. For each group element g∈G, we attach an edge between every $x_{i}$ and $x_{j}$ such that $x_{j}=g\cdot x_{i}$. Edges can thus be unambigously indexed by $\left(x_{i},g\right)$. While this graph is presented here as a purely combinatorial construction, we note that this graph can be viewed as a categorical object called the action groupoid [8]. The coboundary operator d of the multigraph (interpreted as a cellular complex) sends vertex functions to an edge-valued function. Since the graph is complete, for any edge $\left(x_{i},g\right)$, the image of the coboundary operator evaluated at that edge is $df\left(x_{i},g\right)=f\left(x_{j}\right)-f\left(x_{i}\right)=f\left(g\cdot x_{i}\right)-f\left(x_{j}\right)$. Vertex functions in the kernel of the coboundary operator corresponds to functions that satisfy f(g·x)=f(x). Since the Laplacian is defined as $L=d^{†}d$, we leave it as an exercise for the reader to verify that the Laplacian is proportional to L in Equation (A44) up to a constant.*

*In the more general case where χ(g) is unitary for all g, operator L can be interpreted as a cellular sheaf graph Laplacian [9], following a similar construction.*
